# Hydrogen Sulfide-Induced Vasodilation: The Involvement of Vascular Potassium Channels

**DOI:** 10.3389/fphar.2022.911704

**Published:** 2022-06-01

**Authors:** Xiao-Yu Liu, Ling-Ling Qian, Ru-Xing Wang

**Affiliations:** Department of Cardiology, Wuxi People’s Hospital Affiliated to Nanjing Medical University, Wuxi, China

**Keywords:** hydrogen sulfide, vasodilation, ATP-sensitive potassium channels, Ca^2+^-activated potassium channels, voltage-dependent potassium channels

## Abstract

Hydrogen sulfide (H_2_S) has been highlighted as an important gasotransmitter in mammals. A growing number of studies have indicated that H_2_S plays a key role in the pathophysiology of vascular diseases and physiological vascular homeostasis. Alteration in H_2_S biogenesis has been reported in a variety of vascular diseases and H_2_S supplementation exerts effects of vasodilation. Accumulating evidence has shown vascular potassium channels activation is involved in H_2_S-induced vasodilation. This review aimed to summarize and discuss the role of H_2_S in the regulation of vascular tone, especially by interaction with different vascular potassium channels and the underlying mechanisms.

## Introduction

Hydrogen sulfide (H_2_S) has been recognized as the third gasotransmitter following nitric oxide and carbon monoxide. Endogenous H_2_S is mainly produced by enzyme pathways *in vivo*, including cystathionine γ-lyase (CES), cystathionine β-synthase (CBS), cysteine aminotransferase and 3-mercaptopyruvate sulfurtransferase (3-MST) (Olas, 2015). In the vascular system, H_2_S is principally synthesized by CES (Szabo, 2012). Biogenesis of H_2_S or downregulation of H_2_S pathway has been found in a variety of vascular diseases, such as hypertension, atherosclerosis, diabetes and pulmonary hypertension (Barton and Meyer, 2019; Dillon et al., 2021; Dorofeyeva et al., 2021; Roubenne et al., 2021). H_2_S plays an important role in regulation vascular homeostasis, such as angiogenesis, proliferation of vascular smooth muscle cells, leukocyte adhesion, oxidative stress and vascular dilation (Wang et al., 2015). The mechanisms underlying H_2_S induced vascular dilation are not completely understood.

Potassium channels are important determinant of vascular tone (Lorigo et al., 2020; Burkhoff et al., 2021). Activation of potassium channels in vascular cells leads to membrane hyperpolarization and subsequent vasodilatation. Five types of potassium channels have been recognized in vascular cells, including voltage-dependent potassium channels (K_V_), Ca^2+^-activated potassium channels (K_Ca_), ATP-sensitive potassium channels (K_ATP_), inward rectifier potassium channels (Kir), and tandem two-pore potassium channels (K_2P_) (Dogan et al., 2019). Accumulating evidence has shown vascular potassium channels activation is involved in H_2_S-induced vasodilation (Li et al., 2021; Marinko et al., 2021; Wen et al., 2021). However, the interaction of H_2_S with different vascular potassium channels and the underlying mechanisms have not been reviewed systematically. This review aimed to summarize and discuss the effects of H_2_S in the regulation of vascular tone, especially by interaction with different vascular potassium channels and the underlying mechanisms.

## Role of Hydrogen Sulfide in Vascular Diseases and Vasodilation

Biogenesis of H_2_S or downregulation of H_2_S has been found in a variety of vascular diseases. Plasma H_2_S levels were significantly lower in ever-treated hypertensive patients compared with normal subjects, and were higher in patients with well-controlled blood pressure than in patients with grade 2 and 3 hypertension, suggesting that the reduction of endogenous H_2_S plays a role in the pathophysiology of hypertension (Sun et al., 2007). The H_2_S generating enzymes, CSE and 3-MPST were significantly reduced in cutaneous tissue of hypertension adults (Greaney et al., 2017); CSE and CBS expression in lymphocytes were reduced of hypertensive patients (Cui et al., 2020). In patients with portal hypertension, plasma H_2_S was significantly lower than that in normal subjects, and the level of H_2_S was negatively correlated with the severity of the disease (Wang et al., 2014). In patients with pulmonary hypertension, plasma level of H_2_S was decreased (Sun et al., 2014) and negatively correlated with pulmonary artery diameter (Liao et al., 2021). The increase of H_2_S predicted lower pulmonary artery diameter (Liao et al., 2021). Thus, H_2_S may be involved in these vascular diseases, providing a new method for the diagnosis and treatment of these vascular diseases.

Accumulating evidence indicated endogenous H_2_S or H_2_S supplementation may prevent the development of the vascular diseases. 8 weeks administration of NaHS, a H_2_S donor, lowered tail artery pressure in spontaneously hypertensive rats (SHR) (Sun et al., 2015). Tain et al. (2018) also reported early short-term NaHS treatment prevented the development of hypertension in SHRs. Exogenous supply of H_2_S reduced pulmonary hypertension (Chunyu et al., 2003). The intravenous injections of H_2_S donors, Na_2_S and NaHS, decreased systemic arterial pressure in the anesthetized rats (Yoo et al., 2015). The predominantly effect of H_2_S or the H_2_S donors on regulation of vascular tone is vasodilation, although H_2_S has vascular constrictive effects in certain conditions (Orlov et al., 2017). NaHS produced vasorelaxation in different vessel beds, including aorta (Köhn et al., 2012), coronary artery (Hedegaard et al., 2014), carotid artery (Centeno et al., 2019), and mesenteric small artery (White et al., 2013). GYY4137, a slow-releasing H_2_S donor, induced concentration-dependent relaxation in rat mesenteric arteries (Abramavicius et al., 2021) and bovine posterior ciliary artery (Chitnis et al., 2013). N-phenylthiourea (PTU) and N, N′-diphenylthiourea (DPTU) compounds, showing long-lasting H_2_S donation, promoted vasodilation in rat aortic rings (Citi et al., 2020). Novel H_2_S donors, AP67 and AP72, also elicited a dose-dependent relaxation in phenylephrine precontracted-bovine posterior ciliary arteries vessels (Kulkarni-Chitnis et al., 2015). L-cysteine, the substrate for CSE, caused a vasodilation in mouse mesenteric artery (Hart, 2020). However, the mechanisms of H_2_S induced-vasodilation are not fully understood. It may be closely related to vascular potassium channels.

## Hydrogen Sulfide-Induced Vasodilation *Via* Activation of Potassium Channels

Potassium channels play important roles in regulating of membrane potential and vascular tone. There are many studies having been carried out on H_2_S-induced vasodilation *via* activation of potassium channels in different vascular beds. Modulation of potassium channel activity by H_2_S donors may be a novel treatment for vascular diseases. This review presents the effects and mechanisms of H_2_S-induced vasodilation *via* activation of potassium channels, including ATP-sensitive potassium channels (K_ATP_), calcium activated potassiumchannels (K_Ca_) and voltage dependent potassium channels (K_V_).

### ATP-Sensitive Potassium Channels

The active K_ATP_ channel structurally is a hetero-octamer of four Kir6 subunits at the center and four sulfonylurea receptor (SUR) subunits surrounded (Li et al., 2017). Kir6 forms the channel pore and SURs are regulatory subunits (Alquisiras-Burgos et al., 2022). Activation of K_ATP_ channels can induce membrane hyperpolarization of the vascular smooth muscle cell, and then lead to the vasodilation.

H_2_S acted as a K_ATP_ channel regulator. Glibenclamide, a K_ATP_ channel blocker, significantly inhibited vasodilation-induced by NaHS in carotid arteries of diabetic rabbits (Centeno et al., 2019). H_2_S was involved in the anticontractile effect of perivascular adiposetissue on aorta of hypertensive pregnant rat and the anticontractile effect was eliminated by K_ATP_ blocker (Souza-Paula et al., 2020). NaHS decreases the myogenic response of cerebral arterioles and this effect was partially mediated by K_ATP_ channels (Liu et al., 2012). Na_2_S-induced relaxation of human umbilical vein was also impaired by glibenclamide (Mohammed et al., 2017). Maximum vadilation of constricted mouse aorta elicited by NaHS was unaffected by removal of the endothelium but significantly attenuated by glibenclamide (Al-Magableh and Hart, 2011). In addition to Na_2_S and NaHS, other H_2_S donors, such as DPTU, caused vasodilation in rat aorta and was abolished by K_ATP_ blocker (Citi et al., 2020). ZYZ-803, a novel H_2_S and NO conjugated donor, relaxed the aortic contraction induced by PE and the effect was suppressed by glibenclamide (Wu et al., 2016). Glibenclamide significantly attenuated the dilation effect induced by AP67 and AP72 (H_2_S donors) (Kulkarni-Chitnis et al., 2015) and GYY4137 (Chitnis et al., 2013) respectively on bovine ciliary arteries. H_2_S plays a role in the vasodilation in response to the reagents, such as moringa oleifera leaves (Aekthammarat et al., 2020) and sulforaphane (Parfenova et al., 2020), and their vasodilation effects was also reduced by glibenclamide. These data suggested an important role of K_ATP_ in H_2_S-induced vasodilation and the possible mechanisms are discussed as below.

First, H_2_S directly activated K_ATP_ channel. H_2_S increased the whole-cell currents and the single channel open probability of K_ATP_ channels. Inhibition of endogenous H_2_S production reduced whole-cell K_ATP_ currents in mesenteric artery smooth muscle cells (Tang et al., 2005). Second, K_ATP_ channel can be sulfhydrated by H_2_S. H_2_S-mediated sulfhydration and hyperpolarization are absent in cells overexpressing C43S mutant Kir 6.1. Mutating the site of sulfhydration (Kir 6.1 C43S) inhibited H_2_S-induced hyperpolarization. Sulfhydration augments K_ATP_ activity by enhancing Kir 6.1-PIP2 binding and reducing Kir 6.1-ATP binding (Mustafa et al., 2011). Third, H_2_S-induced vasodilation by activating K_ATP_ is mediated by its subunits. Liang et al. (2011) reported glibenclamide partially reversed H_2_S induced potassium currents in piglet cerebral arterial smooth muscle cells and H_2_S-induced vasodilation was much smaller in cerebral arteries of SUR2 subunit knockout mice than in wild type mice. Additionaly, H_2_S promoted vasodilation in SHR, partly through upregulating the expression of Kir6.1 and SUR2B subunits of K_ATP_. H_2_S inhibited phosphorylation of forkhead transcription factors FOXO1 and FOXO3a, stimulated their nuclear translocation, and enhanced their binding with Kir6.1 and SUR2B promoters (Sun et al., 2015). However, several studies indicated K_ATP_ channel had no effect in hydrogen sulfide mediated vasodilation, as K_ATP_ blocker did not attenuate H_2_S-induced vasodilation (Streeter et al., 2012; White et al., 2013; Kutz et al., 2015), suggesting other potassium channels are involved in H_2_S-induced vascular regulation.

### Calcium Activated Potassium Channels

According to the conductance, calcium activated potassium channels are divided into three groups: the large conductance calcium-activated potassium channels (BK, 100–300pS), the intermediate conductance calcium-activated potassium channels (IK, 25–100pS) and the small conductance calcium-activated potassium channels (SK, 2–25pS) (Qian et al., 2014). BK channel is a tetramer consist of α-subunit and β-subunit (Salkoff et al., 2006). The β-subunits has four isoforms (β1–β4) and the β1-subunits are primarily expressed in vascular smooth muscle cells (Lee and Cui, 2010; Wu and Marx, 2010).

Iberiotoxin, a BK channel specific inhibitor, reduced maximal relaxation of human saphenous vein induced by NaHS (Marinko et al., 2021), and Na_2_S produced dilation of mouse coronary arteries (Chai et al., 2015). Exogenous H_2_S dilated mesenteric arteries of intermittent hypoxia rats, requires BK channels (Jackson-Weaver et al., 2011). Blockers of BK channel also inhibited GYY4137 induced relaxations in rat mesenteric arteries (Abramavicius et al., 2021).

The activation of BK channel by H_2_S is mainly mediated by the following two mechanisms. First, H_2_S activates BK channels by increasing channel currents. BK current density was 91 ± 42 pA/pF under baseline condition while 201 ± 31 pA/pF after exposure to Na_2_S at testing potential 150 mV in mouse coronary arterial smooth muscle cells (Chai et al., 2015). Second, BK channel was activated by Ca^2+^influx increase induced by H_2_S. Naik et al. (2016) reported Na_2_S increased sulfhydration of transient receptor potential vanilloid type 4 channels in aortic endothelium cells. Activation of transient receptor potential vanilloid type 4 channels by H_2_S caused Ca^2+^ influx, resulting in BK channels activation in endothelial cells and subsequent endothelial hyperpolarization, and vascular dilation. Furthermore, the activation of endothelial BK channels by H₂S can also increase Ca^2+^ sparks in mesenteric artery smooth muscle cells, thus resulted in vasodilation (Jackson-Weaver et al., 2013).

IK and SK channels are also distributed in vascular cells (Liu et al., 2018). H_2_S-induced vasodilation was partly inhibited by selective IK and SK channel inhibitor charybdotoxin and SK channel inhibitor apamin. H_2_S stimulated IK and SK in endothelial cells, leading to hyperpolarization and vasodilation (Mustafa et al., 2011). Cheng et al. (2004) also reported charybdotoxin/apamin-sensitive K^+^ channels in endothelium cells had an important role in H_2_S effect on rat mesenteric artery. The expression of SK2.3 was decrease in CSE knockout mice or by CSE inhibitor, and was upregulated by H₂S (Tang et al., 2013). Expression of IK was enhanced in carotid arteries from diabetic rabbits, and the relaxant action of NaHS was significantly inhibited by charybdotoxin and 4-amynopiridine in diabetic arteries (Centeno et al., 2019). Thus, H₂S-induced vasodilation was partly through IK and SK.

### Voltage Dependent Potassium Channels

Kv channels is a diverse family of outwardly rectifying potassium channels (including K_V1_–K_V12_) (Hua et al., 2022). Each K_V_ channel comprises a α-subunit and an ancillary β-subunit. α-subunits form the ion conducting pore and β-subunits modulate the activity of α-subunits. K_V_ channels are major determinants of membrane potential and play a key role in regulating vascular dilation (Rhee and Rusch, 2018). And the distribution of K_V1_, K_V7_, and K_V9.3_ are reported in vascular smooth muscle cells of different vessel beds (Nieves-Cintrón et al., 2018).

NaHS relaxed carotid arteries precontracted by phenylephrine of diabetic rabbits, while 4-amynopiridine, a K_V_ channel blocker, significantly inhibited the relaxant effect of NaHS, suggesting a role of K_V_ in H_2_S induce vasodilation in diabetes (Centeno et al., 2019). Vasodilation to NaHS was also inhibited by blocking K_V_ channels in mouse mesenteric arteries (Hart, 2020), but only a significant shift to the right of the concentration-response curve of NaHS without affecting maximum dilation in the aorta (Al-Magableh and Hart, 2011).

K_V7_ channel, encoded by KCNQ gene, is thought as a powerful mechanism to H_2_S induced vasorelaxation. NaHS produced concentration-dependent vasorelaxation, which was blocked by XE991, a K_V7_ channel inhibitor. Perivascular adipose tissue released H_2_S, which play a major role in control of vascular tone *via* K_V7_ channel (Schleifenbaum et al., 2010). XE991 also inhibited H₂S-induced vasodilatation of porcine coronary arteries (Hedegaard et al., 2014) and murine aortas (Köhn et al., 2012). GYY4137-induced relaxation was also inhibited by K_V7_ blocker (Abramavicius et al., 2021). The mechanism may be related to persulfidation of K_V7_ channels by CBS-derived H_2_S (Vellecco et al., 2020).

## Conclusion

Taken together, H_2_S is involved in vascular diseases, such as hypertension, diabetes, pulmonary hypertension and so on. H_2_S causes vasodilation in numerous vascular beds and regulates vascular tone by interaction with different vascular potassium channels, including K_ATP_, K_Ca_, and K_V_ channels *via* the following different mechanisms. H_2_S can directly activated vascular K_ATP_ and BK channels and increased sulfhydration of K_ATP_ and K_V7_ channels. H_2_S promoted vasodilation in partly through upregulating the expression of Kir6.1 and SUR2B subunits of K_ATP_, or BK channel activation by increased Ca^2+^influx. Thus, H_2_S may present a potential therapeutic target on vascular diseases. However, the vasorelaxant effect of H_2_S in different vessel beds is complex. More studies are needed to focus on the activation mechanisms of H_2_S on potassium channels, and novel, low-releasing H_2_S donors are required for treatment in the future.
